# Mapping quantitative trait loci and predicting candidate genes for *Striga* resistance in maize using resistance donor line derived from *Zea diploperennis*

**DOI:** 10.3389/fgene.2023.1012460

**Published:** 2023-01-12

**Authors:** B. Badu-Apraku, S. Adewale, A. Paterne, Q. Offornedo, M. Gedil

**Affiliations:** International Institute of Tropical Agriculture (IITA), Ibadan, Nigeria

**Keywords:** *Striga* resistance, QTL x environment, candidate genes, marker assisted selction, wild maize

## Abstract

The parasitic weed, *Striga* is a major biological constraint to cereal production in sub-Saharan Africa (SSA) and threatens food and nutrition security. Two hundred and twenty-three (223) F_2:3_ mapping population involving individuals derived from TZdEI 352 x TZEI 916 were phenotyped for four *Striga*-adaptive traits and genotyped using the Diversity Arrays Technology (DArT) to determine the genomic regions responsible for *Striga* resistance in maize. After removing distorted SNP markers, a genetic linkage map was constructed using 1,918 DArTseq markers which covered 2092.1 cM. Using the inclusive composite interval mapping method in IciMapping, twenty-three QTLs influencing *Striga* resistance traits were identified across four *Striga*-infested environments with five stable QTLs (*qGY*4, *qSC*2.1, *qSC*2.2, *qSC*5, and *qSC*6) detected in more than one environment. The variations explained by the QTLs ranged from 4.1% (*qSD*2.3) to 14.4% (*qSC*7.1). Six QTLs each with significant additive × environment interactions were also identified for grain yield and *Striga* damage. Gene annotation revealed candidate genes underlying the QTLs, including the gene models GRMZM2G077002 and GRMZM2G404973 which encode the GATA transcription factors, GRMZM2G178998 and GRMZM2G134073 encoding the NAC transcription factors, GRMZM2G053868 and GRMZM2G157068 which encode the nitrate transporter protein and GRMZM2G371033 encoding the SBP-transcription factor. These candidate genes play crucial roles in plant growth and developmental processes and defense functions. This study provides further insights into the genetic mechanisms of resistance to *Striga* parasitism in maize. The QTL detected in more than one environment would be useful for further fine-mapping and marker-assisted selection for the development of *Striga* resistant and high-yielding maize cultivars.

## Introduction

Maize is one of the world’s most important food and feed crops and plays an important role in ensuring food security. *Striga hermonthica* (purple witchweed) is an obligate hemiparasitic plant that parasitizes the root systems of cereals, causing significant reduction in maize yield in the savannas of sub-Saharan Africa (SSA). Losses in maize yield due to *Striga* infestation could reach as high as 100%, particularly when infestation is severe during the vegetative growth stage (Akaogu et al., 2019). The severity of infestation is influenced by the type of maize cultivar, weather conditions, and the severity of infestation. The parasitic weed penetrates the roots of maize plants with its haustoria cells to derive nutrients, thus depriving host plants of resources necessary for growth (Parker and Riches, 1993). The increasing *Striga* problems cause an annual loss of US $7–10 billion per annum to the livelihoods of African farmers, and the elevated levels in vast areas further worsen food insecurity, hunger, and poverty for millions of subsistence farmers (Rodenburg et al., 2005; Jamil et al., 2022). Effective control of *S. hermonthica* is essential for food security and poverty alleviation for small-holder subsistence farmers in SSA. Commonly used control methods include chemical, cultural and biological measures, which have achieved little or no success. Under prevailing field conditions in SSA, especially in the savannas, the use of *Striga*-resistant maize varieties has been found to be the most effective, economical, and eco-friendly approach for the control and prevention of yield losses due to *Striga* parasitism (Akaogu et al., 2019). Also, host plant resistance could be deployed as a vital component of an integrated control strategy for mitigating the menace of the parasitic weed. In *Striga* research, a maize plant is characterized as resistant when the parasite is unable to penetrate through the endodermis of the host plant, thus disallowing xylem-xylem connections for continuity after attachment of the plant to the host (Atera et al., 2015), whereas tolerance refers to the extent to which the *Striga* damage effects on the maize plants are mitigated (Rodenburg et al., 2017; Badu-Apraku et al., 2020b).

Quantitative trait loci (QTL) mapping has become an important tool for dissecting the genetic architecture of polygenic traits, facilitating the identification of genomic regions underlying traits of interest, the distribution of gene effects and the relative importance of additive, dominance, and epistatic effects (Vengadessan et al., 2013). With the development of sequencing technology, high-density SNPs are now used in constructing genetic maps for increasing precision (Zhang et al., 2022). Several QTL studies have been conducted to understand the molecular genetics of complex traits under both biotic and abiotic stresses in maize, including drought (Tuberosa et al., 2002; Almeida et al., 2013), low soil nitrogen (Ribeiro et al., 2018), maize grey leaf spot disease (Berger et al., 2014), *Fusarium* ear rot (Maschietto et al., 2017), maize streak virus (Ladejobi et al., 2018), and *Striga* parasitism (Amusan et al., 2008; Badu-Apraku et al., 2020a; Badu-Apraku et al., 2020b). However, information is limited on the molecular genetics of *Striga* resistance. The use of marker-assisted selection (MAS) could be a very effective strategy in breeding for *Striga* resistance/tolerance (Badu-Apraku and Fakorede, 2017). Molecular markers associated with *Striga* resistance alleles would be invaluable because plant breeders could use such markers during selection to identify the resistance loci in existing populations or to pyramid resistance into new populations. However, the success of MAS depends on the identification of the accurate locations of the QTL and the identification of tightly linked molecular markers.

Wild relatives of maize (teosintes and *Tripsacum dactyloides*) have been used to develop genetically improved maize with resistance to *Striga* species, which are particularly prevalent in Africa (Rich and Ejeta, 2008). Novel resistance genes identified in the wild perennial maize relative, teosinte (*Zea diploperennis*) by International Institute of Tropical Agriculture (IITA) maize breeders (Kling et al., 2000) have been introgressed into early and extra-early maturing maize inbred lines (Amegbor et al., 2017). For example, the early-maturing *Striga* resistant and drought-tolerant maize inbred line, TZdEI 352 derived from a biparental cross involving the normal endosperm white maize population TZEW Pop DT STR and the *Z. diploperennis* has displayed increased grain yield and durable *Striga* resistance/tolerance (Akaogu et al., 2019). Results of genetic studies conducted by Akaogu et al. (2019) revealed that the additive-dominance model was adequate in describing observed variations in the number of emerged *Striga* plants in a population derived from a cross between TZdEI 352 and the susceptible inbred TZEEI 11. Hence, the digenic epistatic model was adopted for the inheritance of resistance to *Striga* damage (Akaogu et al., 2019). In contrast, dominance effects were higher than the additive effects for the number of emerged *Striga* plants across locations signifying that non-additive gene action conditioned the inheritance of *Striga* resistance. It was proposed that the inbred TZdEI 352 could serve as an invaluable parent for hybrid development in *Striga* endemic agro-ecologies of SSA. Furthermore, TZdEI 352 has significant and positive general combining ability (GCA) effects for grain yield in *Striga,* drought and low N environments, significant negative GCA effects for the stay-green characteristic under drought and low N, as well as negative and significant GCA effects for *Striga* damage and number of emerged *Striga* plants under *Striga*-infested conditions. Inbred TZdEI 352 is presently serving as an invaluable multiple stress tolerant parent for hybrid development in *Striga* endemic agro-ecologies of SSA. This inbred line was therefore an ideal genotype for QTL discovery for *Striga* resistance. Application of molecular markers provides a powerful tool for mapping quantitative traits loci and improving complex traits through marker-assisted selection (MAS). QTLs/markers have been identified for *Striga* resistance traits by maize breeders at the IITA and the International Maize and Wheat Improvement Centre (CIMMYT). However, most of the QTLs/markers have minor effects, though linked to genes associated with plant defense mechanisms under *Striga* infestation (Badu-Apraku et al., 2020a; Adewale et al., 2020; Badu-Apraku et al., 2020b; Gowda et al., 2021; Stanley et al., 2021). There is, therefore, the need to identify more *Striga* resistance QTLs particularly using germplasm extracted from wild maize sources (*Zea diploperennis*) to ensure successful resistance breeding through gene pyramiding. The objectives of this study were to (i) map QTLs for *Striga* resistance using a maize population derived from a cross involving the *Striga* resistant early maturing inbred, TZdEI 352, and the *Striga* susceptible early maturing line, TZEI 916, (ii) identify candidate genes associated with the *Striga* resistance QTLs.

## Materials and methods

### Plant materials

The contrasting inbred lines used in the present study were selected based on preliminary evaluation of the lines (Adewale et al., 2020) and genetic studies involving these inbred lines under *Striga*-infested environments at Mokwa, Nigeria in 2017 and 2018. The two early maturing white maize inbred lines varied significantly in their responses under artificial *Striga* infestation. The parental inbred line TZdEI 352 (TZE-W POP STR 107 S6 24/254-1/2-1/1-1/1-2/2-1/1) is *Striga* resistant, while line TZE1 916 ((TZE COMP 5-W DT C7 x TZEI 31) S6 inb 39-1/1-1/2-2/2-2/2-1/1) is highly susceptible to *Striga* parasitism. The inbred TZEI 916 has negative and significant GCA effect for grain yield, positive and significant GCA effect for *Striga* damage as well as positive GCA effect for number of emerged *Striga* counts under *Striga* infestation. The *Striga* resistant parent TZdEI 352 also possesses drought and low soil N tolerance alleles and it is one of the parental lines involved in the development of the outstanding multiple stress-tolerant hybrid (HAKIMI 3) commercialized in Nigeria. It is also involved in several other hybrids and open-pollinated varieties in the pipeline for release in several countries in West and Central Africa. Crosses were made between TZdEI 352 and TZEI 916 to obtain 223 F_2:3_ families at the IITA-Ibadan breeding nursery during the 2018 and 2019 growing seasons. The inbred TZdEI 352 was crossed to TZEI 916 to obtain F_1_ progenies. The F_1_ progenies derived from the bi-parental cross involving TZdEI 352 and TZEI 916 were screened using SSR molecular markers to identify true-to-type hybrids for the development of the F_2_ mapping population. Thirty-four F_1_ progenies identified to be true-to-type were advanced to the F_2_ stage through selfing. The harvested F_2_ seeds were planted for sample collection for genotyping and advancement to the F_2:3_ stage for field phenotyping. The F_2:3_ mapping population was used to identify QTL/genomic regions responsible for resistance/tolerance to *Striga* parasitism.

### Field experiment and trait evaluation

A segregating population consisting of 223 F_2:3_ families and the two parental lines (TZdEI 352 and TZEI 916) were evaluated at two *Striga* endemic locations: Mokwa (9^0^18′N, 5^0^4′E, 457 m altitude) and Abuja (9^0^ 16′N, 7^0^ 20′E, 300 m altitude) in the Southern Guinea savanna zones of Nigeria. Field trials were conducted during the 2019 and 2020 growing seasons at each location. The experimental design used in the different environments was 15 x 15 lattice design. The experimental field at Mokwa has a luvisol soil type, while that at Abuja has a ferric luvisol soil type (Soil Survey Staff, 1999). The trials were replicated twice for evaluations in each of the four environments. Each experimental unit consisted of a 1-row plot 3 m long, with row spacing of .75 m and intra-row spacing of .4 m. The fields for artificial *Striga* infestation at Mokwa and Abuja were injected with ethylene gas about 2 weeks before planting to induce suicidal germination of *Striga* seeds in the soil. The artificial *Striga* infestation was carried out as proposed by the IITA Maize Program (Kim, 1991). About a week before inoculation, the *Striga* seeds were thoroughly mixed with finely sieved sand at the ratio of 1:99 by weight to ensure rapid and uniform infestation. A standard scoop calibrated to deliver about 5,000 germinable *Striga* seeds per hole was used for the artificial infestation. Three maize seeds were placed in the same hole with the *Striga* seeds and thinned to two plants per hill at 2 weeks after emergence to obtain the target population density of 66,666 plants per ha. Fertilizer application on the maize plots was delayed till about 30 days after planting to subject the maize plants to stress, a condition that was expected to favour the production of strigolactones, which enhances good germination of *Striga* seeds and attachment of *Striga* plants to the roots of host plants. At this stage of plant growth, 30 kg N/ha, and 30 kg each of P and K were applied as NPK 15-15-15. The reduced rate of fertilizer application was necessary because *Striga* emergence decreases at high N rate (Kim, 1991). Data were recorded on the number of emerged *Striga* plants and host plant damage severity at 10 weeks after planting (Kim, 1991; Badu-Apraku et al., 2020b). The number of ears per plant (EPP) was computed by dividing the total number of ears harvested per plot by the number of plants in a plot at harvest. Grain yield (kg ha^− 1^) was estimated from field weight of ears harvested per plot, assuming a shelling percentage of 80% (that is, 800 g grain/kg ear weight), adjusted to moisture content of 15%. These four traits measured (grain yield, host plant damage, number of emerged *Striga* plants and ears per plant) are the primary traits of interest in selecting for resistance to *Striga* parasitism in maize (Menkir and Kling, 2007; Adewale et al., 2020).

### Statistical analysis of phenotypic data

Statistical analysis of the phenotypic data was performed using the SAS software version 9.3 and R software version 4.0.5. Analysis of variance (ANOVA) was first carried out for each environment. Thereafter, combined ANOVA across environments (locations) was conducted with PROC GLM in SAS using a random statement with the TEST option (SAS Institute, 2011). The replications and blocks within replications were considered as random and the F_2:3_ families as fixed effects. Correlation analysis among the traits was carried out using package “corrplot” in the R software. The broad sense heritability (H^2^) was estimated using the formula: $H^{2}=\sigma g^{2}/\left(\sigma g^{2}+\sigma {е}^{2}/r\right)$ where $\sigma g^{2}$ is the genetic variance; $\sigma {е}^{2}$ is the residual error and r = number of replications. The best linear unbiased estimates (BLUEs) were estimated for each genotype under each and across environments using the mixed linear model (MLM) in META-R software (Alvarado et al., 2016).

### DNA extraction, genotyping and data filtering process

Young, healthy leaf tissues were obtained from the F_2:3_ individuals and bulk samples from the parental lines. The samples were freeze-dried and used for genomic DNA extraction following the DArT protocol. The extracted DNA was tested by electrophoresis in 1% agarose gel and analyzed on the ND-1000 spectrophotometer platform (NanoDrop, Wilmington, DE, United States) for concentration and purity. The mapping population was genotyped using 15,048 DArTseq markers for the QTL identification. Low-quality data filtering was carried out by eliminating markers with minor allele frequency of less than 5%, missing data greater than 10%, and those that were monomorphic for the parents. Segregation distortion was determined using Chi-square (χ2) test for goodness-of-fit and an expected ratio of 1:2:1 for the F_2:3_ mapping population. Markers significantly deviating (*p* < .05) from Mendelian segregation were eliminated. After quality filtering, a total of 1,918 quality SNP markers were used for the QTL analysis.

### Construction of genetic linkage map, QTL analysis and candidate gene annotation

Recombination fractions and LOD scores were performed using the R qtl package (http://www.rqtl.org) and the order of the markers were clustered into bin. The correct order of the markers across each chromosome was verified through the pairwise marker linkage analysis using the “est.rf” function. Genetic linkage maps were then constructed using R/QTL 2. The genetic distances were estimated using the “est.map” function with “kosambi” distance from the R/qtl package (Broman et al., 2003; Arends et al., 2010). Quantitative trait loci analysis was performed using the inclusive composite interval mapping (ICIM-ADD) method in IciMapping V4.2 (Meng et al., 2015). The ICIM approach utilizes a strategy in which a stepwise regression is firstly performed, so markers with significant effect on QTL are selected. ICIM-ADD method was used with the multi-environmental model built in QTL IciMapping (Li et al., 2015; Singhal et al., 2021). The parameters of QTL analysis were set as follows: logarithm of odds (LOD) = 1,000 permutations, step = 1 cM, and PIN = .001. The confidence interval of each QTL was determined by LOD > 3. The software also estimated the proportion of phenotypic variation, additive and dominance effects explained by each QTL for a trait. QTL × environment interaction (QEI) mapping for multi-environmental trials (MET) was carried out using ICIM-ADD (Meng et al., 2015; Wang et al., 2022). Sources of favourable alleles were determined depending on signs of the QTL additive effects and the traits of interest. For instance, positive additive effects for grain yield and ears per plant and negative additive effects for *Striga* damage and *Striga* emergence counts indicated that the favourable alleles were derived from the resistant parent and *vice versa*. The QTLs detected in more than one environment were regarded as stable (Shang et al., 2015). QTL were classified as major when the phenotypic variance was more than 10% and minor when less than 10% (Collard et al., 2005). QTL detected under different environments for the same trait were regarded as the same QTL when the distances of their peaks were less than 10 cM (Ma et al., 2018; Zhang et al., 2020). QTL naming conventions was followed as described by McCouch et al. (1997) and Yang et al. (2020). The name of each QTL was defined starting with a lowercase ‘*q*’, then the trait name in uppercase, thereafter by the chromosome number where the QTL was detected. For instance, *qSD2* refers to a QTL identified for *Striga* damage (SD) on chromosome 2. Candidate genes within the main additive QTLs confidence intervals and their corresponding molecular functions were retrieved using the B73 reference genome (Woodhouse et al., 2021). The candidate genes were mined from the sequences of the identified *QTL* related to the plant defense functions.

## Results

### Phenotypic evaluation

**FIGURE 1:**
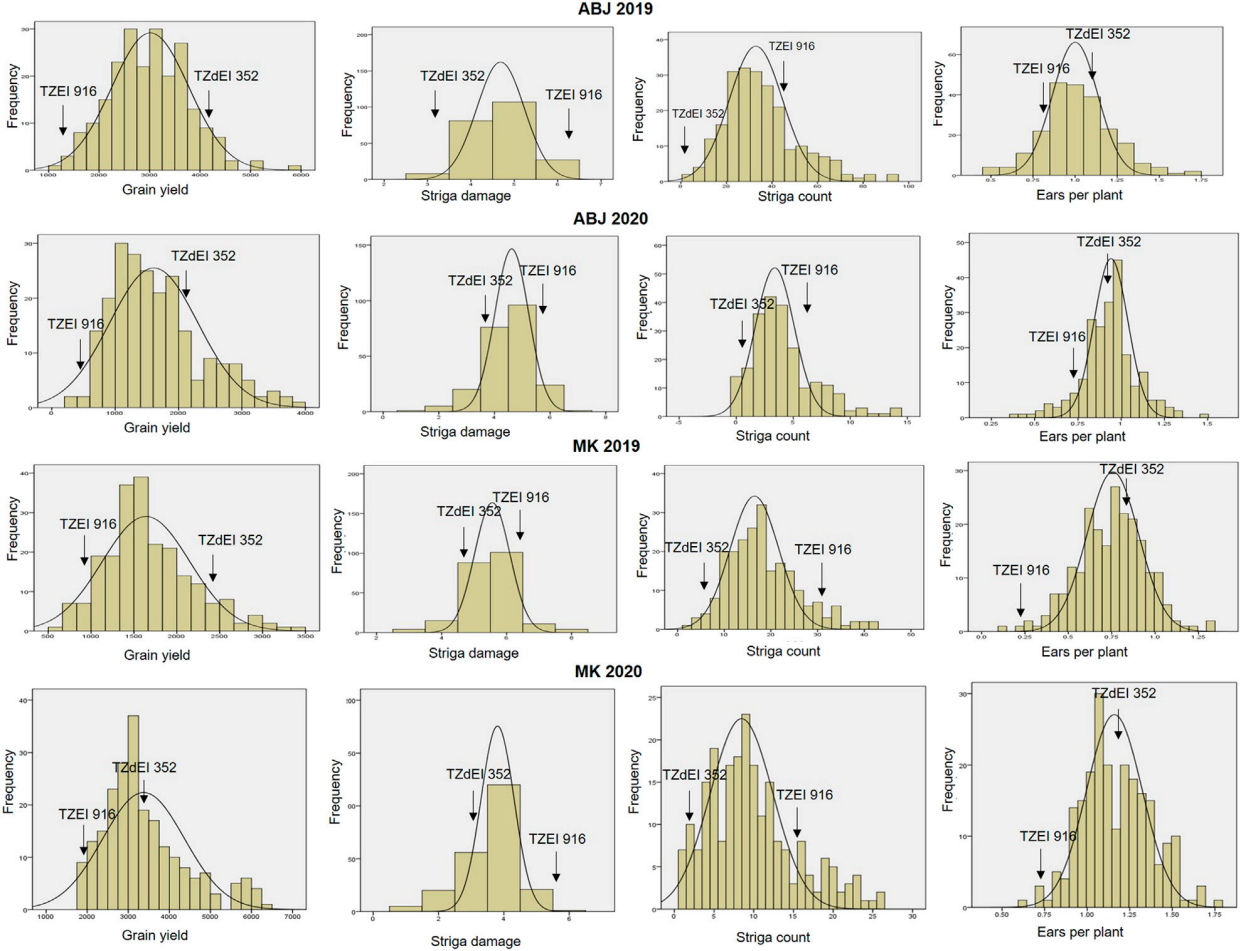
Frequency distribution of grain yield, number of ears per plant, *Striga* damage and number of emerged *Striga* plants among the F_2:3_ mapping population individuals, 2019–2020. ABJ—Abuja, MK—Mokwa.

**FIGURE 2:**
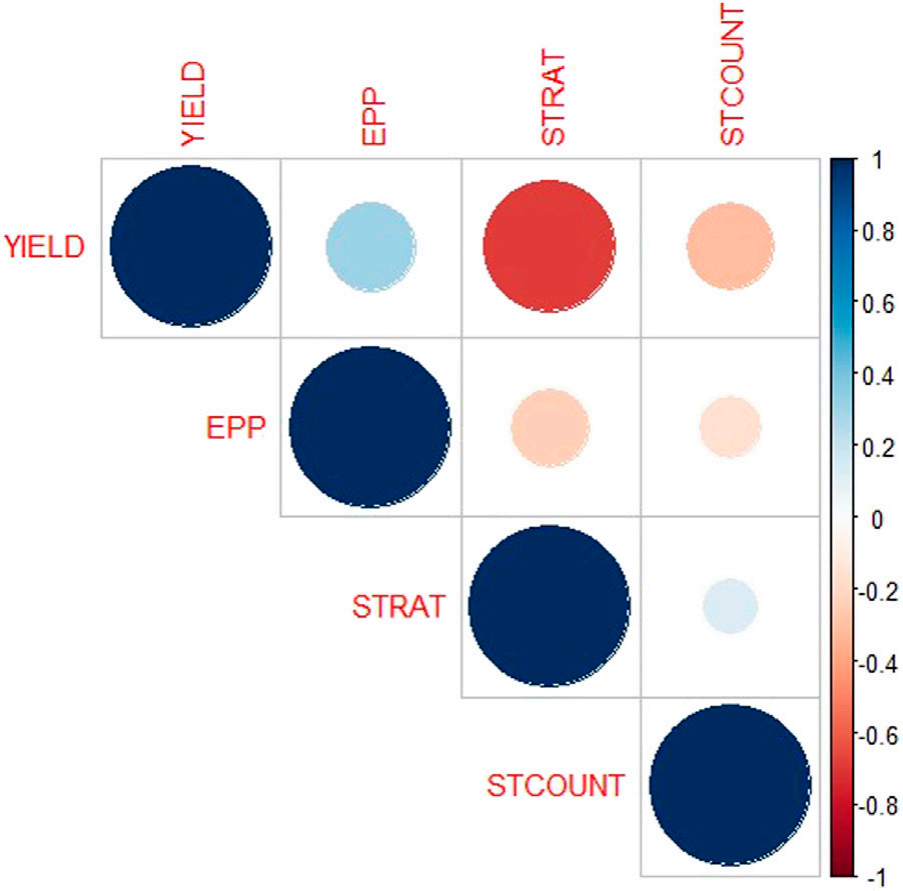
Relationship among *Striga* adaptive traits in the F_2:3_ mapping population across four *Striga*-infested environments. YIELD—Grain yield, EPP—number of ears per plant, STRAT—*Striga* damage rating, STCOUNT—*Striga* emergence counts. Gradient bars represent the correlation coefficients.

The analysis of variance showed significant variation among the 223 F_2:3_ families and their parents for grain yield and all other *Striga* adaptive traits across artificial *Striga*-infested environments (Table 1). Significant effects of the environment and genotype × environment interaction were observed for measured traits except genotype × environment interaction for *Striga* emergence counts. Similarly, the parents and the F_2:3_ individuals displayed high level of variability for the *Striga* resistance adaptive traits in each environment (Table 2; Figure 1). All the traits followed normal distribution and both absolute values of skewness and kurtosis were less than 1.0. Transgressive segregation was observed in both directions of normal distribution for the studied traits. Among the F_2:3_ individuals, grain yield varied from 317 kg/ha to 5,380 kg/ha, with the smallest mean of 1,646, largest mean of 3,388 and largest standard deviation of 1245.39. Ears per plant differed from .01 to .95 with the smallest mean of .74, largest mean of 1.17 and largest standard deviation of .30, *Striga* damage varied from 1 to 9 with the smallest mean of 3.6, largest mean of 5.5 and largest standard deviation of 1.17, while *Striga* count differed from 0 to 4.96 with the smallest mean of 1.25, largest mean of 3.35 and largest standard deviation of .90. Broad-sense heritability of the traits ranged from .33 for ears per plant to .60 for grain yield. Results of correlation analysis among the *Striga* adaptive traits across the four research environments revealed strong negative association of grain yield with *Striga* damage rating and *Striga* emergence counts but significant positive relationship with ears per plant (Figure 2). Ears per plant displayed negative association with *Striga* damage rating and *Striga* emergence counts. *Striga* damage rating also revealed positive association with *Striga* emergence counts.

**TABLE 1:** Mean squares of F_2:3_ mapping population evaluated under artificial *Striga* infestation across four environments at Abuja and Mokwa in the 2019 and 2020 growing seasons.

Source of variation	Df	Grain yield	Ears per plant	Striga damage 2	Striga count 2
Block (Rep*Env)	112	1920007**	.09**	2.30**	1.50**
Rep (Env)	4	9991537**	.67**	25.08**	8.95**
Env	3	364246330**	14.07**	266.14**	366.92**
Genotype	224	2037509**	.09**	1.64**	.79**
Genotype x Env	672	823112**	.06**	.98**	.35
Error	784	466939	.05	.67	.34
H^2^		.60	.33	.42	.52

**TABLE 2:** Summary statistics of the parental lines and F_2:3_ individuals under artificial *Striga* infestation.

Trait	ENV	Parents		F_2:3_ population							
		TZdEI 352	TZEI 916	Mean	SD	Min	Max	CV (%)	Skew	Kurt	H^2^
Yield	AB19	4177.63	1166.19	3,000.63	1115.33	1193.43	5,911.80	17.17	−.15	−.57	.52
	AB20	2110.12	420.01	1646.34	900.77	272.13	3,817.00	14.71	.56	−.31	.59
	MK19	2430.13	919.98	1675.59	783.52	593.75	3,382.66	16.76	.57	−.22	.37
	MK20	3,477.53	1934.08	3,388.88	1245.39	1763.89	6263.58	21.75	.59	−.18	.74
EPP	AB19	1.11	.81	1.02	.27	.50	1.70	16.24	.02	.62	.46
	AB20	.93	.64	.93	.22	.38	1.50	13.36	−.61	.77	.32
	MK19	.83	.23	.74	.30	.12	1.32	20.70	.00	−.31	.30
	MK20	1.16	.74	1.17	.27	.69	1.90	15.75	−.12	−.51	.33
RAT	AB19	3.34	6.32	4.70	1.03	3.00	6.00	22.03	.10	.24	.38
	AB20	3.34	5.61	4.48	1.22	1.00	7.00	17.34	−.59	.57	.33
	MK19	4.50	6.45	5.46	1.17	3.00	8.00	21.47	−.12	.36	.48
	MK20	3.74	5.61	3.60	.96	1.00	6.00	16.65	−.44	.23	.57
CO	AB19	.13	43.48	35.45	25.87	3.00	93.00	22.97	−.19	−.48	.44
	AB20	.58	5.88	4.10	4.63	.00	14.00	29.09	.02	−.90	.34
	MK19	5.87	31.16	17.99	11.06	2.00	42.00	21.45	−.40	.81	.44
	MK20	2.67	15.40	9.87	8.54	1.00	26.00	26.53	−.12	−.51	.42

AB19—Abuja 2019, AB20—Abuja 2020, MK19—Mokwa 2019, MK20—Mokwa 2020.

### Linkage map

**FIGURE 3:**
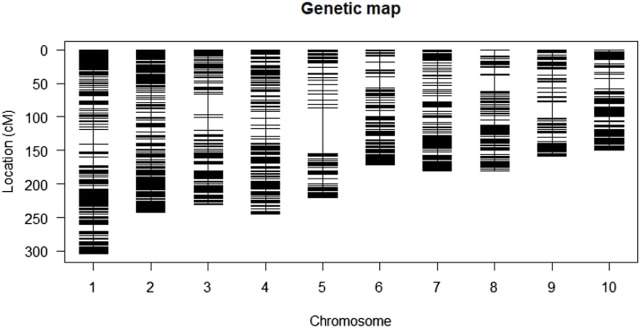
Genetic map constructed using DArT markers from the F_2:3_ mapping population.

The genetic map of the F_2:3_ population contained 1,918 polymorphic markers distributed across the 10 maize chromosomes with a total coverage length of 2092.1 cM. The length of each chromosome varied from 150 cM for chromosome 10–307 cM for chromosome 1 (Table 3; Figure 3). The highest number of markers (306) was recorded on chromosome 1, whereas chromosome 5 recorded the least number of markers (117). The average interval size was 23.3 cM. The mean linkage group length was 209.21 cM with a mean of 191.9 cM loci. The genetic linkage map of the F_2:3_ mapping population displaying stable QTLs as well as QTLs similar to those reported in previous studies are presented in Supplementary Figure S1. The order of markers on the genetic map conforms to the physical position of the marker and is also supported by pairwise marker linkage analysis (Supplementary Figure S2).

**TABLE 3:** DArT markers linkage map of F_2:3_ mapping population derived from the cross TZdEI 352 x TZEI 916.

Linkage group	Number of markers	Map length (cM)	Max gap (cM)
Chr1	306	306.9	22.1
Chr2	303	244.3	8.35
Chr3	212	231.8	31.9
Chr4	198	245.1	11.15
Chr5	117	222	68.1
Chr6	173	172	17.6
Chr7	179	180.7	9.2
Chr8	161	181	25.1
Chr9	119	158.4	24.6
Chr10	150	149.9	15.24
Total	1918	2092.1	233.34
Average per linkage group	191.8	209.21	23.334

### QTL identification for *Striga* resistance adaptive traits

QTL mapping was carried out using the inclusive composite interval mapping method to identify QTLs for *Striga* resistance traits and phenotypic BLUE values across environments were used to reduce the variation effects of the environments. A total of 23 QTLs were detected for the *Striga* adaptive traits in the F_2:3_ population across four environments (that is, Abuja in 2019 and 2020; Mokwa in 2019 and 2020) (Table 4). Five stable QTLs including one for grain yield (*qGY*4), and four for *Striga* emergence counts (*qSC*2.1, *qSC*2.2, *qSC*5 and *qSC*6), were detected in more than one environment. For grain yield, four QTLs *qGY*2, *qGY*4, *qGY*5 and *qGY*8 were detected on chromosomes 2, 4, 5, and 8, respectively. The LOD scores of these QTLs ranged from 4.0 to 6.2, and the proportion of phenotypic variance explained (PVE) ranged from 8.2% to 11.6%. The highest PVE of 11.6% was recorded by *qGY*5. The QTLs *qGY*2 and *qGY*8 displayed negative additive effects, whereas *qGY*4 and *qGY*5 displayed positive additive effects for grain yield. Most of the QTLs identified across environments were also found in individual environments. For instance, the QTL *qGY*2 was also detected in MK19, *qGY*4 both in AB19 and MK20, *qGY*5 in MK20 and *qGY8* in AB20. Eight QTLs *qSD*2.1, *qSD*2.2, *qSD*2.3, *qSD*3.1, *qSD*3.2, *qSD*5, *qSD*8 and *qSD*10 were identified for *Striga* damage on chromosomes 2, 3, 5, 8 and 10 across the four environments. These QTLs individually explained 4.1%–10.2% phenotypic variation, with LOD scores ranging from 2.6 to 3.9. The highest PVE of 10.2% was recorded by *qSD*5. Of the eight QTLs identified for *Striga* damage across environments, only *qSD*2.2, *qSD*3.1, *qSD*3.2, and *qSD*5 displayed negative additive effects. The QTL *qSD*2.2 was also found in individual environment AB20; *qSD*2.3 and *qSD*3.1 were also identified in AB19. Nine QTLs *qSC*2.1, *qSC*2.2, *qSC*3, *qSC*5, *qSC*6, *qSC*7.1, *qSC*7.2, *qSC*8 and *qSC*9 were identified for number of emerged *Striga* counts on chromosomes 2, 3, 5, 6, 7, 8, and 9 across environments. The LOD scores of the identified QTLs varied from 3.0 to 5.4, with PVE ranging from 4.6% to 14.4%. The QTL *qSC*7.1 recorded the highest PVE of 14.4%. Of the nine QTLs, only *qSC*2.2, *qSC*3, *qSC*5, and *qSC*7.2 displayed negative additive effects. The QTLs *qSC*2.1, *qSC*2.2, *qSC*5, *qSC*7.1, *qSC*8 and *qSC*9 were also identified in AB19, *qSC*2.1, *qSC*2.2 and *qSC*3 were identified in MK19, and *qSC*2.1 in MK20. Two QTLs qEP1 and qEP6 were identified on chromosomes 1 and 6 for number of ears per plant. LOD score of 2.7 and PVE of 7.0% were obtained for qEP1, while qEP7 recorded LOD score of 3.9 and PVE of 10.3%. Pleiotropic effects were observed for *qGY*5 and *qSD*5 located on the same position on chromosome 5 as well as *qSD*3 and *qSC*3 found within the same confidence interval on chromosome 3. Significant G × E interaction effects were identified for all studied traits except *Striga* emergence counts. Using the MET functionality of the Ici-QTL mapping software, six MET-QTLs each were identified for grain yield and *Striga* damage as well as one for ears per plant (Table 5). Three MET-QTLs (e*qGY*-2, e*qGY*-5 and e*qGY*-8) detected for grain yield were located close to the main additive effects QTLs (*qGY*-2, *qGY*-5 and *qGY*-8). Similarly, the MET-QTLs (e*qSD*2.1) is closely located to the main additive effects QTL (*qSD*2.1) for *Striga* damage. These MET-QTLs displayed the same signs of additive effects as the main effects QTLs.

**TABLE 4:** Quantitative trait loci identified from F_2:3_ mapping population derived from TZdEI 352 x TZEI 916 across four artificial *Striga* infested environments in Nigeria, 2019-2020.

Trait	QTL^#^	QTL-IND	Chr	Peak position	LOD	PVE (%)	Add	Dom	LeftCI	RightCI	References	QTL-MI^a^
Grain yield	*qGY2*	MK19	2	23.8	4.03	8.2	−159.29	115.64	22.3	25.3		
	*qGY4*	AB19, MK20	4	230.8	5.45	10.8	219.62	−122.50	228.3	232.3		
	*qGY5*	MK20	5	76.8	6.15	11.6	235.60	−17.41	72.3	80.3		
	*qGY8*	AB20	8	168.1	4.48	8.7	−125.21	234.76	167.6	168.6	Badu-Apraku et al. (2020b)	167.2–176.6
*Striga* damage	*qSD2.1*		2	172.8	3.35	7.4	.13	−.18	171.3	173.3		
	*qSD2.2*	AB20	2	181.2	3.83	8.9	−.17	.04	180.3	186.2		
	*qSD2.3*	AB19	2	201.8	2.80	4.1	.11	−.10	199.3	202.3	Stanley et al. (2021)	188.1
	*qSD3.1*	AB19	3	13.9	2.89	5.3	−.09	.16	10.4	15.4		
	*qSD3.2*		3	168.9	3.79	6.8	−.17	.00	168.4	169.4		
	*qSD5*		5	76.8	3.86	10.2	−.18	.01	70.3	80.3	Stanley et al. (2021)	70.4
	*qSD8*		8	164.1	3.49	8.6	.07	−.23	161.6	164.6		
	*qSD10*		10	110.7	2.55	5.9	.17	−.06	109.2	112.2	Adewale et al. (2020); Okunlola et al. (2022)	112.7
*Striga* count	*qSC2.1*	AB19, ABJ20, MK19	2	12.8	4.84	8.0	2.89	−2.09	12.3	13.3	Stanley et al. (2021)	13.5
	*qSC2.2*	AB19, MK19	2	215.8	5.40	12.6	−.14	.07	215.3	216.3	Stanley et al. (2021)	209.0
	*qSC3*	MK19	3	11.9	3.36	4.6	−.01	.17	11.4	13.4		
	*qSC5*	AB19, MK20	5	212.8	2.96	6.1	−.10	.06	211.3	214.3		
	*qSC6*	AB19, MK20	6	110.0	3.26	7.0	2.81	−.12	107.5	110.5		
	*qSC7.1*	AB19	7	41.8	3.17	14.4	4.73	−2.59	40.3	42.3		
	*qSC7.2*		7	109.8	3.70	7.1	−.14	.02	101.3	111.3		
	*qSC8*	AB19	8	166.1	3.65	7.3	.13	−.02	165.6	166.6		
	*qSC9*	AB19	9	14.2	5.13	11.3	.17	.04	13.7	16.7		
Ears per plant	*qEPP1*		1	46.8	2.72	6.0	−.03	.03	43.3	52.3		
	*qEPP6*		6	.96	3.87	8.3	−.04	−.01	.96	1.5		

^a^ Marker interval/position of QTLs, in previous studies, QTL^#^- QTL, detected across four *Striga-*infested environments; QTL-IND, Individual environments where the QTLs, were also detected, AB19—Environment Abuja 2019, AB20 - Environment Abuja 2020, MK19 - Environment Mokwa 2019, MK20 - Environment Abuja 2020.

**TABLE 5:** Characteristics of QTLs related to *Striga* adaptive traits detected using QEI mapping.

Trait	MET-QTL	Chr	Pos (cM)	Left marker	Right marker	LOD	LOD (A)	LOD (A x E)	PVE	PVE(A)	PVE (A x E)	ADD	QTL × environment interaction			
													A x E1	A x E2	A x E3	A x E4
Yield	e*qGY*-2	2	21.81	Chr2_20.599078	Chr2_21.824408	12.97	6.22	6.76	4.49	4.17	2.32	−152.75	110.72	−63.76	85.95	−132.91
	e*qGY*-3	3	141.85	Chr3_141.751814	Chr3_142.030105	13.32	4.67	8.65	7.32	6.96	3.36	65.41	−156.72	−16.50	−109.38	282.60
	e*qGY*-4	4	240.83	Chr4_238.180488	Chr4_241.595508	10.56	4.70	5.86	3.46	1.63	1.83	−88.53	56.37	−77.27	71.95	−51.04
	e*qGY*-5	5	81.76	Chr5_77.117931	Chr5_82.996929	11.26	6.46	4.80	7.30	6.86	2.44	−166.86	−23.68	−53.14	172.06	−95.23
	e*qGY*-7	7	160.85	Chr7_160.472394	Chr7_161.599627	13.44	6.03	7.41	6.64	5.09	3.55	67.08	−81.83	49.43	−170.57	202.97
	e*qGY*-8	8	171.08	Chr8_170.7423	Chr8_171.423574	10.56	4.70	5.86	3.46	1.63	1.83	−142.40	63.08	−70.91	38.10	−30.27
*Striga* damage	e*qSD*-2.1	2	176.81	Chr2_176.643883	Chr2_178.099007	6.28	1.38	4.90	4.32	.48	.84	.10	.08	−.17	.22	−.13
	e*qSD*-2.2	2	6.81	Chr2_6.742061	Chr2_7.400568	8.34	3.34	5.00	4.06	3.14	2.92	−.01	.06	.04	.11	−.21
	e*qSD*-3	3	176.85	Chr3_172.349067	Chr3_181.958432	5.16	3.89	1.27	2.39	1.25	1.14	−.11	−.06	−.27	.13	.20
	e*qSD*-5	5	46.76	Chr5_43.565479	Chr5_47.752516	5.50	2.61	2.89	5.23	3.44	1.79	.14	−.12	.02	.03	.07
	e*qSD*-7	7	110.85	Chr7_109.161107	Chr7_111.241692	5.45	2.11	3.34	6.09	4.10	1.99	−.04	−.11	.07	.11	−.08
	e*qSD*-10	10	5.70	Chr10_5.605598	Chr10_5.755017	5.45	2.11	3.34	5.09	3.10	1.99	.08	−.18	.17	−.08	.10
Ears per plant	e*qEPP*-6	6	110.96	Chr6_110.501667	Chr6_112.722037	12.73	.03	7.70	3.08	2.20	.88	.01	−.11	.01	.08	.02

### Candidate gene prediction

Based on the 23 main additive effect QTLs detected for four *Striga* adaptive traits using the QTL mapping, a total of 279 protein coding genes were identified within the confidence interval of each of the identified QTLs (Supplementary Table S3), according to the maize gene annotation database accessible at Maize GDB (https://www.maizegdb.org). Of these, 36 candidate genes were functionally annotated to be associated with the *Striga* resistance QTLs detected (Table 6). Inside the putative QTL *qGY*2 is enclosed the gene GRMZM2G475678, ereb61 (locus–2:21824408-24239657) which encodes the ethylene-responsive transcription factor 61. Other genes enclosed in this QTL include GRMZM5G805505, GRMZM2G077002, GRMZM2G178998, GRMZM2G055180, GRMZM2G123387, GRMZM2G404973, and GRMZM2G054795 which encode AP2-EREBP-transcription factor 87, C2C2-GATA-transcription factor 5, NAC-transcription factor 131, EREBP-transcription factor 198, WRKY-transcription factor 101, C2C2-GATA-transcription factor 11 and C2C2-YABBY-transcription factor 1 respectively. On chromosome 4, one putative QTL *qGY*4 (locus - 4:228266384-232427685) contained three genes GRMZM2G085751, GRMZM2G164359 and GRMZM2G052102 which encodes the bHLH-transcription factor 24, C3H-transcription factor 343 - putative RING zinc finger domain superfamily protein and bZIP-transcription factor 120 - basic leucine zipper 19, respectively. Similarly, the QTL *qGY*8 (locus - 8:167208138-168572930) located .47 Mb upstream of the QTL peak possesses the gene models GRMZM2G030762, GRMZM2G065971 which encodes the bHLH-transcription factor 55 and magnesium transporter 8. Inside the pleiotropic QTLs *qGY* 5 and *qSD* 5 (locus 5:68355981-77117931) located on chromosome 5, are five genes cdpk22, nrt3, myb161, bhlh129 and bzip14. These genes encode calcium-dependent kinase, nitrate transporter, MYB, bHLH as well as the bZIP-transcription factors, respectively. The QTLs *qSD*3.1 and *qSC*3 (locus 3:11798990-14131442) as well as *qSD*2 (locus 2:201773609-203194405) were associated with the MYB transcription factors. On chromosome 10, QTL *qSD*10 located 2.2 Mb downstream of the QTL peak contains the genes GRMZM2G425798, GRMZM2G313756 and GRMZM2G023708 encoding the AP2-EREBP-transcription factor 149, bhlh100 - bHLH-transcription factor 100 and AP2-EREBP-transcription factor 125. The QTL *qSC*2 (locus 2:12757631–13953770) which encodes the genes GRMZM2G024898, GRMZM2G038722, GRMZM2G014534 and GRMZM2G055204 were associated with WRKY-transcription factor 70, MYB20 transcription factor 13, MYB-related-transcription factor 36 and AP2-EREBP-transcription factor 18, respectively. Similarly, QTLs *qSC*2 (locus 2:215446898-2159609600), *qSC*7 (locus 7:39974564-43809836) and *qEPP*1 (locus 1:43333475–46957478) which houses the genes GRMZM2G383841, GRMZM2G075715 and GRMZM2G021095 encodes the bHLH-transcription factor 147, ARF-transcription factor 37 as well as LBD-transcription factor 4, respectively. On chromosome 5, *qSC*5 (locus 5:211699547–213923453) contains genes GRMZM2G130459 and GRMZM2G036092 encoding the AP2-EREBP-transcription factor 187 and bHLH-transcription factor 30. The QTL *qSC*8 (locus 8:165139590–166861798), around .76 Mb upstream region of the QTL peak contains the genes GRMZM2G134073 and GRMZM2G371033 encodes the NAC and SBP transcription factors, respectively. On chromosome 9, QTL *qSC*9 (locus 9 9:13152043–14927006) contains genes GRMZM2G156006 and GRMZM2G402156 which encode the AP2-EREBP-transcription factor 207 and MYB-related-transcription factor 19, respectively.

**TABLE 6:** Key candidate genes associated with QTLs detected from the F_2:3_ mapping population evaluated under artificial *Striga* infestation.

Trait	QTL	LG:start-end position	Gene ID	Predicted function of candidate gene
Grain yield	*qGY*2	2:21824408-24239657	GRMZM2G475678,ereb61	ethylene-responsive transcription factor ERF061
			GRMZM5G805505,ereb87	AP2-EREBP-transcription factor 87
			GRMZM2G077002,gata5	gata5—C2C2-GATA-transcription factor 5
			GRMZM2G178998,nactf131	nactf131—NAC-transcription factor 131
			GRMZM2G055180,ereb198	AP2-EREBP-transcription factor 198
			GRMZM2G123387,wrky101	wrky101—WRKY-transcription factor 101
			GRMZM2G404973,gata11	gata11—C2C2-GATA-transcription factor 11
			GRMZM2G054795,yab1	yab1—C2C2-YABBY-transcription factor 1
	*qGY*4	4:228266384-232427685	GRMZM2G085751,bhlh24	bhlh24—bHLH-transcription factor 24
			GRMZM2G164359,c3h43	c3h43—C3H-transcription factor 343—putative RING zinc finger domain superfamily protein
			GRMZM2G052102,bzip120	bzip120—bZIP-transcription factor 120—basic leucine zipper 19
	*qGY*8	8:167208138-168572930	GRMZM2G030762,bhlh55	bhlh55—bHLH-transcription factor 55
			GRMZM2G065971,mgt8	mgt8—magnesium transporter8
Yield; *Striga* damage	*qGY*5, *qSD*5	5:68355981-77117931	GRMZM2G053868, GRMZM2G157068,cdpk22	cdpk22—calcium dependent protein kinase22
			GRMZM2G163866,nrt3	nrt3—nitrate transport3
			GRMZM2G088189,myb161	myb161—MYB-transcription factor 161
			GRMZM5G856837,bhlh129	bhlh129 - bHLH-transcription factor 129
			GRMZM2G153144,bzip14	bzip14—bZIP-transcription factor 14
*Striga* damage rating	*qSD*2	2:201773609–203194405	GRMZM2G050305, GRMZM5G892094,myb31	myb31—MYB31 transcription factor31
	*qSD*10	10:108463928–113256953	GRMZM2G425798,ereb149	AP2-EREBP-transcription factor 149
			GRMZM2G313756,bhlh100	bhlh100—bHLH-transcription factor 100
			GRMZM2G023708,ereb125	AP2-EREBP-transcription factor 125
*Striga* damage, *Striga* emergence	*qSD*3.1, *qSC*3	3:11798990–14131442	GRMZM2G052377,myb20	myb20—MYB20 transcription factor20
*Striga* emergence count	*qSC*2.1	2:12757631–13953770	GRMZM2G024898,wrky70	wrky70—WRKY-transcription factor 70
			GRMZM2G038722,myb13	myb13—MYB20 transcription factor13
			GRMZM2G014534,mybr36	mybr36—MYB-related-transcription factor 36
			GRMZM2G055204,ereb18	ereb18—AP2-EREBP-transcription factor 18
	*qSC*2.2	2:215446898–215960960	GRMZM2G383841,bhlh147	bhlh147—bHLH-transcription factor 147
	*qSC*5	5:211699547–213923453	GRMZM2G130459,ereb187	AP2-EREBP-transcription factor 187
			GRMZM2G036092,bhlh30	bhlh30—bHLH-transcription factor 30
	*qSC*7	7:39974564–43809836	GRMZM2G075715,arftf37	arftf37—ARF-transcription factor 37
	*qSC*8	8:165139590–166861798	GRMZM2G134073,nactf9	nactf9—NAC-transcription factor 9
			GRMZM2G371033,sbp18	sbp18—SBP-transcription factor 18
	*qSC*9	9:13152043–14927006	GRMZM2G156006,ereb207	ereb207—AP2-EREBP-transcription factor 207
			GRMZM2G402156,mybr19	mybr19—MYB-related-transcription factor 19
Ears per plant	*qEPP*1	1:43333475–46957478	GRMZM2G021095,lbd4	lbd4—LBD-transcription factor 4

## Discussion

The introgression of novel genes for *Striga* resistance from wild relative of maize, *Zea diploperennis* L. into other genetic backgrounds using molecular markers has great potential for the development of high-yielding and *Striga* resistant maize genotypes. The significant differences observed among individuals of the F_2:3_ mapping population for measured traits revealed high level of variability and the suitability for QTL mapping analysis. Frequency distribution and box plot analysis revealed that the studied traits were normally distributed in the F_2:3_ mapping population. Skewness and kurtosis for the four measured traits were less than 1.0 indicating normal distribution of traits and the suitability of the data for QTL analysis. Some of the F_2:3_ individuals displayed phenotypes which were beyond the parental limits indicating the possible role of transgressive segregation and additive genes from the two parental lines (Mondal and Badigannavar, 2019). Transgressive segregation was observed in both directions of normal distribution for the studied traits. The presence of transgressive segregation suggested genetic recombination, implying that both favourable and unfavourable alleles for the traits were distributed between the parents. Transgressive segregation observed for measured traits also suggested the possible existence of multiple QTLs and QTL × QTL interaction or epistatic interaction (Chattopadhyay et al., 2019).

In bi-parental mapping, the detection of robust QTLs for traits of interest requires significant differences between the two parents. The *Striga* resistant parent TZdEI 352 and the *Striga* susceptible parent TZEI 916 used in our study varied significantly in terms of grain yield, *Striga* damage, *Striga* emergence count and number of ears per plant. The wide range of variation in the present population revealed the possibility for selection of lines with resistance to *Striga* and increased grain yield in maize. The genetic map of the F_2:3_ population contained 1,918 polymorphic markers distributed across the 10 maize chromosomes with a total coverage length of 2092.1 cM. The overall coverage length reported in the present study are similar to those reported by Badu-Apraku et al. (2020a) and Badu-Apraku et al. (2020b), who found total coverage lengths of 2016 cM and 2076 cM, respectively, in studies focusing on the identification of QTLs for *Striga* resistance in two extra-early maturing maize mapping populations. Similarly, Yang et al. (2020) reported total genome coverage length of 2,315 cM across the 10 maize chromosomes using 119 polymorphic simple sequence repeats pairs.

The results of QTL mapping rely on the type of population, nature of traits, number of samples, marker density and QTL mapping techniques used. Good understanding of these factors is important in designing QTL mapping experiments and determining optimal procedures for data analysis especially when dealing with highly polygenic traits such as *Striga* resistance (Su et al., 2017). In the present study, we identified a total of 23 QTLs for grain yield, number of ears per plant, number of emerged *Striga* plants and *Striga* damage ratings in a population involving 223 F_2:3_ families derived from the maize biparental cross TZdEI 352 x TZEI 916 across four artificial *Striga*-infested environments. The QTLs *qGY*5 and *qSD*5 showed co-localization of pleiotropic effects. These QTLs accounted for phenotypic variations ranging from 4.1% (*qSD*2.3) to 14.4% (*qSC*7.1), indicating the complex nature of the *Striga* resistance traits studied. Five (*qGY*4, *qSC*2.1, *qSC*2.2, *qSC*5 and *qSC*6) of these QTLs were detected in more than one environment. The QTL identified in more than one environment would be useful for further fine-mapping and marker assisted selection. Some of the QTLs identified in this study have been reported in previous studies. For example, QTLs *qSD*2.3 and *qSD*5 detected for *Striga* damage, *qSC*2.1 and *qSC*2.2 for *Striga* emergence counts in this study have been previously reported by Stanley et al. (2021). The authors identified significant SNP markers S5_70442824 and S2_188120710 to be associated with *Striga* damage ratings as well as S2_135038935 and S2_208978140 associated with number of emerged *Striga* plants in a genome-wide association study to detect SNP markers linked with *Striga* resistance traits in late maturing maize germplasm. Similarly, the QTL *qGY*-8 has been previously reported by Badu-Apraku et al. (2020a); *qSD*-10 by Adewale et al. (2020) and Okunlola et al. (2022). Six major effect QTLs *qGY*4, *qGY*5, *qSD*5, *qSC*2.2, qsc7.1 and qsc9 were identified on chromosomes 2, 4, 5, 7, and 9, explaining phenotypic variance varying from 10.2% to 14.4%. These QTLs are promising for the introgression of favorable alleles to improve *Striga* resistance in maize through marker-assisted selection. Identification of stable genomic regions inside QTLs will serve as guides in the selection of traits more efficiently. However, genotype × environment interaction QTLs are also important as they significantly influence the total phenotypic variance and additive effect of the main effect QTL located inside or close to them. In our study we detected significant genotype × environment interaction for grain yield, *Striga* damage and number of ears per plant. Similar signs of additive effects observed for some of the MET-QTLs and main effect QTLs revealed that these MET-QTLs had positive effects on the total additive value of the *Striga* resistance adaptive traits. This implied that these MET-QTLs had positive effect on the *Striga* resistance alleles which improved the phenotypic expression resulting in increased grain yield and reduced *Striga* damage under *Striga* infestation.

The QTL *qGY*2 associated with grain yield is linked to gene models GRMZM2G077002 and GRMZM2G404973, C2C2-GATA-transcription factors. The GATA transcription factors are widely involved in the regulation of plant developmental and growth processes, including seed germination, development, light-mediated signalling, and regulation of plant nitrogen metabolism (An et al., 2020). GATA motifs have been detected in regulatory regions of many genes involved in nitrogen assimilation, such as nitrate reductase, nitrite reductase, and glutamine synthetase (Hudson et al., 2011). Therefore, GATA transcription factors play a potential role in coordinating nutrient assimilation and vegetative growth. Similarly, the QTLs *qGY*2 and *qSC*8 were identified to be linked with the gene models GRMZM2G178998 and GRMZM2G134073, respectively (NAC transcription factors). The NAC transcription factors play important roles in the regulation of transcriptional reprogramming associated with plant stress responses (Nuruzzaman et al., 2013). Genes in the NAC family regulate a wide range of developmental processes, including seed development, embryo development, shoot apical meristem formation, fiber development, and cell division. The NAC transcription proteins participate in the regulation of immune signalling pathways to execute critical functions in plant immunity (Yuan et al., 2019). When plants are attacked by pathogens, they perceive the pathogen-derived signals and often activate an efficient and complicated but fine-tuned network of defense hormone-mediated signalling pathways (Peng et al., 2018; Yuan et al., 2019).

The QTLs *qGY*5 and *qSD*5 found to be associated with the gene models GRMZM2G053868 and GRMZM2G157068 both encode the nitrate transporter protein. Nitrate is a major source of N in higher plants. A large proportion of NO_3_ acquired by plants from the soil is actively transported through NO_3_ transporters (NRTs) (Bai et al., 2013). To cope with low or high nitrate concentrations in the soil, plant roots have developed high-affinity and low-affinity nitrate uptake systems involving certain nitrate transporter genes. The nitrate transporter (NRT3) proteins are partner proteins that interact with most NRT2 proteins and contribute to high-affinity nitrate uptake (Guo et al., 2020). Thus, the NRT2 genes combine with the NRT3 genes to enable plants to cope with variable nitrate supplies, thereby improving nitrogen use efficiency. Adewale et al. (2020) in a genome-wide association study to identify markers linked to *Striga* resistance in early maturing tropical maize, found marker SNP S10_133224759 close to the functional gene GRMZM2G164743 (bin 10.05), which encodes an ammonium transporter protein (amt5). Further studies on the nitrate transporter candidate genes could help to understand the regulatory mechanism of *Striga* resistance in the roots of maize plants and determine their usefulness in selecting genotypes with resistance to *Striga* parasitism.

The QTL *qSC*8 associated with *Striga* emergence count was found to be linked with the gene model, GRMZM2G371033, SBP-transcription factor. The SBP-transcription factors are very important for plant growth, development, and defense response as they regulate the specific downstream gene expression, such as the phase transition from vegetative to reproductive stage, leaf development, plant hormone signaling, toxin resistance, copper deficiency response, temperature, and drought stress tolerance (Jiang et al., 2021). The gene model GRMZM2G075715, ARF-transcription factor was associated with the QTL *qSC*7 identified for *Striga* emergence count. Auxin response transcription factors play key roles in plant development, particularly in the regulation of gene expression (Saidi and Hajibarat, 2020; Pratt and Zhang, 2021). Auxin is a plant hormone involved in various stages of plant growth and development, such as apical dominance, tropisms, and vascular patterning. Auxin plays key roles in cell division and cell expansion during the developmental stages and in regulation of a variety of physiological processes including lateral root initiation and shoot elongation (Di et al., 2016). The QTL *qEPP*1 associated with ears per plant was found to be linked with the candidate gene GRMZM2G021095, LBD-transcription factor. The lateral organ boundaries domain (LBD) genes, are plant-specific transcription factor family which play crucial roles in controlling plant architecture, regulating lateral organ development, morphogenesis, and stress tolerance in plants (Majer and Hochholdinger, 2011; Wang et al., 2021). The LBD genes are expressed in a band of cells at the adaxial base of all lateral organs formed from the shoot apical meristem and at the base of lateral roots. The LBD genes also regulate plant cell wall thickening and secondary growth (Chen et al., 2017).

Several well-studied plant transcription factor families, including ethylene response factor (ERF), basic-domain leucine-zipper protein (bZIP), MYB, WRKY, NAC, and members of the basic helix-loop-helix (bHLH) transcription factors associated with defense responses in plants were identified in our study to be associated with *Striga* resistance adaptive traits. Stanley et al. (2021) identified the bHLH transcription factors, putative leucine-rich repeat protein and bZIP transcription factors in a GWAS to identify *Striga* resistance linked markers using the IITA intermediate and late maturing maize inbred lines under artificial *Striga* infestation. Similarly; Gowda et al. (2021) found the bHLH transcription factors to be associated with *Striga* resistance traits in maize. These transcription factor families regulate gene expression in response to a range of biotic stimuli, including microbes (fungi, oomycetes, bacteria) and insects, and downstream defense signalling hormones such as salicylic acid, jasmonic acid, and ethylene (Thatcher et al., 2012). The key candidate genes identified in our study should be further validated to ascertain their influence in improving resistance to *Striga* parasitism in tropical maize germplasm.

## Conclusion

The results of this study further elucidated the understanding and deployment of molecular markers in *Striga* resistance breeding. Twenty-three QTLs were detected for four traits associated with *Striga* resistance across four different environments at two locations over 2 years using the F_2:3_ mapping population. The phenotypic variance explained by each QTL ranged from 4.1% to 14.4%. The QTL *qSC*2.1 was consistently detected in three individual environments and across environments. Our results further revealed the genetic basis of *Striga* resistance traits in maize and will be useful for the marker-assisted selection of *Striga*-resistant maize genotypes, laying the foundation for the fine mapping and cloning of the gene underlying the stable QTLs identified in more than one environment in this study.

## Data Availability

The raw data used/analyzed in this manuscript will be made available by the authors upon request.
