# Supplementary figures and images for: Mapping quantitative trait loci and predicting candidate genes for Striga resistance in maize using resistance donor line derived from Zea diploperennis
Source: Front Genet. 2023 Jan 12;14:1012460. doi: 10.3389/fgene.2023.1012460 (PMC9877281; doi:10.3389/fgene.2023.1012460)

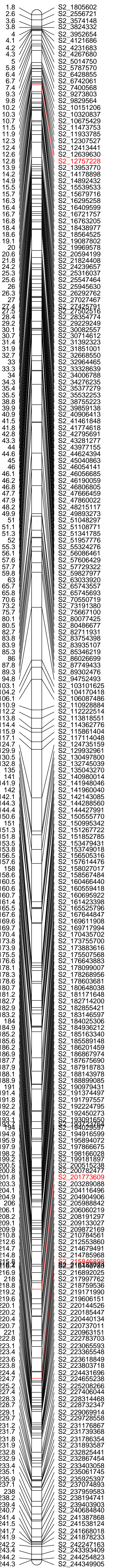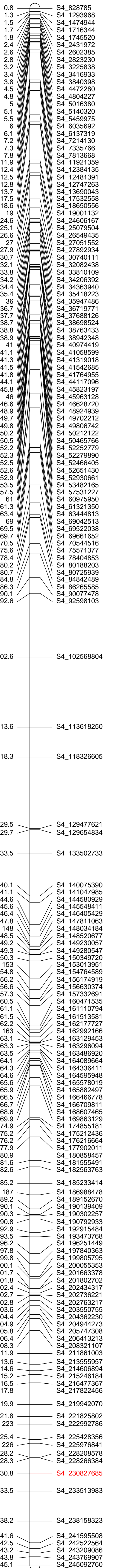

Supplement: Supplementary file 1 [file DataSheet2.PDF]

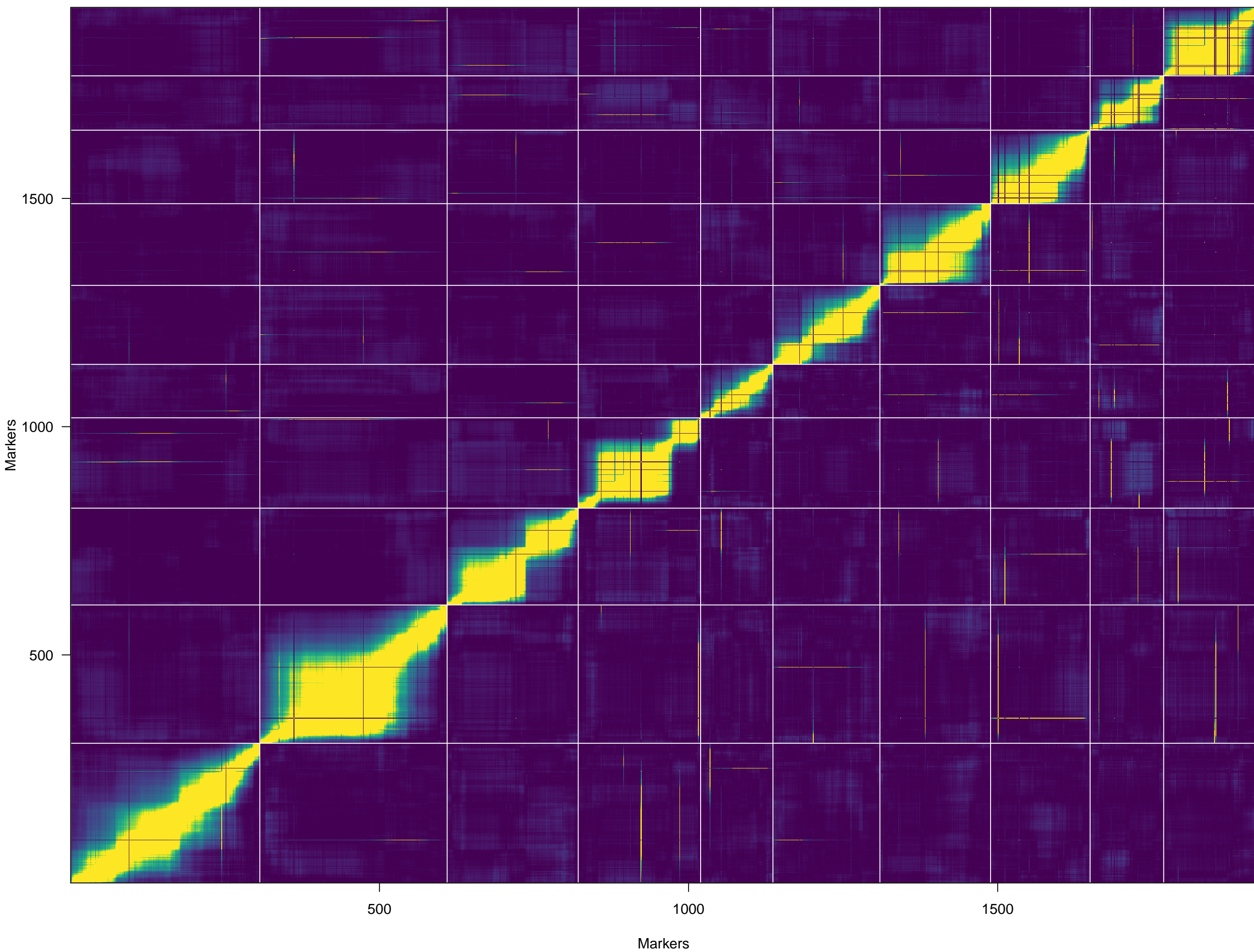

Supplement: Supplementary file 2 [file DataSheet1.PDF]
